# Inflammatory microRNAs in gastric mucosa are modulated by *Helicobacter pylori* infection and proton-pump inhibitors but not by aspirin or NSAIDs

**DOI:** 10.1371/journal.pone.0249282

**Published:** 2021-04-15

**Authors:** Riccardo Vasapolli, Marino Venerito, Wiebke Schirrmeister, Cosima Thon, Jochen Weigt, Thomas Wex, Peter Malfertheiner, Alexander Link

**Affiliations:** 1 Department of Gastroenterology, Hepatology and Infectious Diseases, Otto-von-Guericke University Hospital, Magdeburg, Germany; 2 Department of Internal Medicine II, Hospital of the Ludwig Maximilians University of Munich, Munich, Germany; 3 Medical Laboratory for Clinical Chemistry, Microbiology and Infectious Diseases, Department of Molecular Genetics, Magdeburg, Germany; Institut de Pharmacologie Moleculaire et Cellulaire, FRANCE

## Abstract

Gastric carcinogenesis is associated with alterations of microRNAs (miRNAs) and reversal of these alterations may be a crucial element in cancer prevention. Here we evaluate the influence of *H*. *pylori* eradication, low-dose aspirin (LDA), non-steroidal anti-inflammatory drugs (NSAIDs) and proton-pump inhibitors (PPI) on modification of inflammatory mucosal miRNAs miR-155 and miR-223 in *Helicobacter pylori*-infected and non-infected subjects. The study was performed in two parts: 1) interventional study in 20 healthy subjects with and without *H*. *pylori* infection or following eradication (each n = 10) where LDA (100 mg) was given daily for 7 days; 2) prospective case-control observational study (n = 188). MiR-155 and miR-223 expression was strongly linked to *H*. *pylori*-infection and in short-term view showed a trend for reversal after eradication. Daily LDA as well as regular NSAIDs showed no influence on miRNAs expression both in healthy subjects and patients, while regular PPI intake was associated with lower miR-155 expression in antrum of patients with chronic gastritis independent of density of neutrophils and mononuclear infiltrate. In summary, PPI but not LDA or NSAIDs were associated with modification of inflammatory miRNAs miR-155 and miR-223 in an *H*. *pylori* dependent manner. The functional role of inflammatory miR-155 and miR-223 in understanding of *H*. *pylori*-related diseases needs further evaluation.

## Introduction

Gastric carcinogenesis consists of a complex multistep multifactorial process related to gender, age, dietary habits, and genetic susceptibility factors as well as environmental influences and microbiota. *Helicobacter pylori (H*. *pylori)*-gastritis is a key factor associated with gastric cancer development [1]. *H*. *pylori* colonizes the gastric mucosa, inducing a chronic active gastritis that, in a small percentage of cases, eventually progresses to gastric cancer. According to the Correa´s cascade [2], ongoing chronic non-atrophic gastritis (CNAG) may result in atrophic gastritis (AG) and intestinal metaplasia (IM) in a minority of subjects, in which intraepithelial neoplasia (previously called dysplasia) and invasive gastric cancer may eventually develop. Lauren classified gastric cancer in two subtypes: the intestinal type, that develops usually over decades following classical Correa´s cascade, and the diffuse type, which has a distinct Correa´s cascade-independent molecular pathway of carcinogenesis [3]. Thus, at least in the case of the intestinal type, preventive interventions prior to the development of advanced preneoplastic conditions (AG and IM) are recommended [4, 5].

Accumulating evidence suggests that miRNAs are differentially expressed in gastric cancer and patients with *H*. *pylori* gastritis [6, 7]. We have recently shown that miR-155 and miR-223 are strongly upregulated in gastric mucosa in a stepwise manner in correlation with Correa´s cascade [6, 8]. Latest studies demonstrate a close association between miR-155 and the inflammatory response to *H*. *pylori*-infection, which leads to an increased susceptibility to gastric preneoplastic lesions development [9]. In a mice model, the miR-155 knock-out mice showed a distinct immunological phenotype with a reduced rate of *H*. *pylori* dependent AG and IM development in comparison to a wild type, suggesting that miRNA modification may have a potential therapeutic applicability in gastric cancer prevention [10]. Expression of miR-223 in gastric mucosa and circulating miR-223 are increased in *H*. *pylori*-infected subjects, as well as in patients with gastric cancer [11–13]. In addition, alterations of miR-223 expression have been recently associated with resistance to some chemotherapeutic agents and with promotion of cancer cell proliferation and migration in *H*. *pylori*-related gastric cancer [14, 15].

Non-steroidal anti-inflammatory drugs (NSAIDs) and aspirin (ASA) are frequently used drugs exerting their analgesic, antipyretic and antithrombotic effects through inhibition of cyclooxygenase (COX) activity. The COX-2 isoenzyme is frequently upregulated in inflammation-related carcinogenesis [16, 17]. Evidence from *in vivo* animal studies and epidemiological studies suggests that both NSAIDs and ASA may have anti-tumorigenic properties [18]. Indeed, daily use of these drugs has been associated with reduced occurrence of several gastrointestinal cancers, including colorectal, pancreatic, esophageal and gastric cancers, making them potentially suitable for chemoprevention of such malignancies, at least in high-risk population [19, 20]. Regular intake of low-dose ASA (LDA), for instance, has been associated with a lower risk of non-cardiac gastric cancer among Caucasians [21], whereas long-term Celecoxib (COX-2 selective NSAID) intake may reduce the progression rate or even reverse IM after *H*. *pylori* eradication [22].

Proton pump inhibitors (PPI) are commonly prescribed for prevention and treatment of gastric-acid-related conditions such as peptic ulcer disease and gastroesophageal reflux disease [23]. Previous *in vitro* studies have described a potential anti-inflammatory effect of PPI [24]. Nevertheless, the mechanisms through which PPI exert anti-inflammatory and possibly anti-tumorigenic effects are largely unknown.

In our previous work we have shown that mucosal miR-155 and miR-223 strongly correlate with a mucosal disease state. Whether certain interventions such NSAID-, LDA- or PPI-intake may influence miRNA expression is known. In this work, we studied whether *H*. *pylori*-eradication, NSAID-, LDA- and PPI-intake influenced the expression pattern of the inflammatory miRNAs miR-155 and miR-223 in the gastric mucosa of healthy subjects with and without *H*. *pylori*- gastritis and in patients with various gastric pathologies using a prospective interventional and a case-controls studies, respectively.

## Material and methods

### Study design and study population

The study population comprised of two cohorts: 1) prospective interventional study in healthy subjects with primary aim to evaluate the effect of LDA on gastric mucosa [25]; and 2) prospective case-control study in consecutive patients undergoing upper GI-endoscopy in single tertiary center [26]. Both studies were approved by the Ethics Committee of the Otto-von-Guericke University Magdeburg and were conducted in accordance with the “World Medical association Declaration of Helsinki—Ethical Principles for Medical Research Involving Human Subjects” (143/99 and 80/11) [27]. Written informed consent was obtained from every participant in both studies.

### Interventional study in healthy subjects

**Fig 1:**
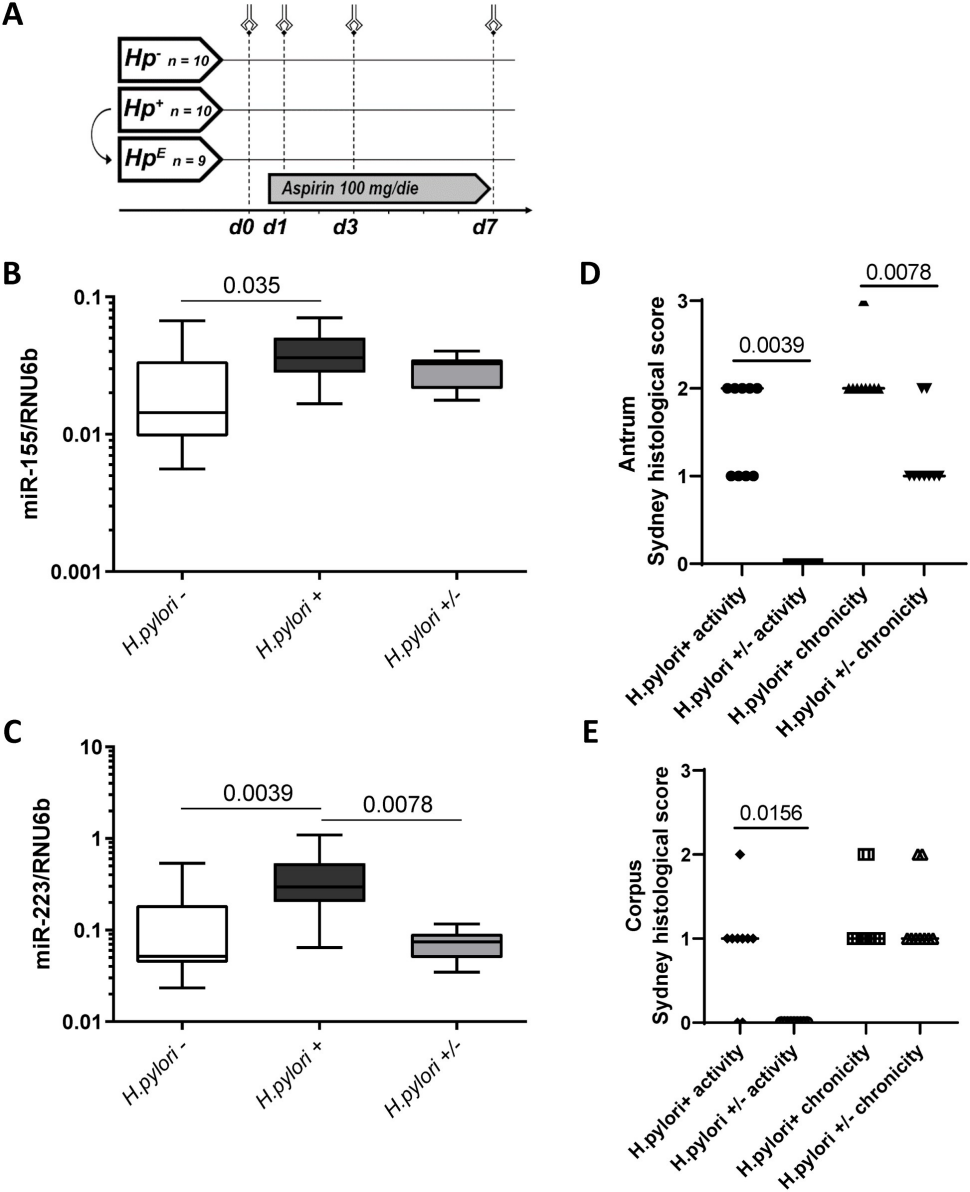
Expression of miR-155 and miR-223 in the context of *H*. *pylori*-gastritis. **(A)** Schematic representation of the LDA-study design (interventional study in healthy subjects); Hp^-^ = *H*. *pylori*-negative group (n = 10); Hp^+^ = *H*. *pylori*-positive group (n = 10); Hp^E^ = *H*. *pylori*-eradicated group (n = 9); volunteers were challenged with a daily dose of LDA (100 mg/die) for 7 days (d1-d7). **B-C**: MiR-155 **(B)** and miR-223 **(C)** expression levels in gastric antral mucosa of *H*. *pylori*-negative healthy individuals (*H*. *pylori*-; n = 10), *H*. *pylori*-infected subjects (*H*. *pylori* +; n = 10) and in individuals after eradication treatment (*H*. *pylori* +/-; n = 9). MiRNA expression levels are shown as 2ΔCT-values normalized to RNU6b and data are shown as box-plots where the horizontal line marks the median value. **D-E**: Histological changes in inflammation following *H*. *pylori* eradication using updated Sydney classification. The data shown as activity and chronicity scores for matching subjects before (*H*. *pylori*+) and 3 months after (*H*. *pylori+/-*) *H*. *pylori* eradication for Antrum **(D)** and Corpus **(E)**. Wilcoxon test was used for statistical analysis.

In order to evaluate the influence of daily LDA intake on gastric mucosa, twenty healthy volunteers (14 males, 6 females; age 31 ± 7 years, *H*. *pylori* positive n = 10 and *H*. *pylori* negative n = 10) were enrolled at the Department of Gastroenterology, Hepatology, and Infectious Diseases of the Otto-von-Guericke University (Magdeburg, Germany) [25]. All *H*. *pylori*-positive participants received eradication therapy (confirmed by breath test) and 9 of them agreed to repeat the protocol after 3 months (*H*. *pylori*-eradicated group). All volunteers received a daily dose of LDA (100 mg) for 7 days. On day 0 (before starting LDA exposition) and at days 1, 3, 7, an esophagogastroduodenoscopy was performed and mucosal biopsies from antrum were taken to perform histological and molecular analyses. Study design is shown in Fig 1A.

### Observational case-control study in patients

The second study population constituted of a total 188 patients that underwent upper GI endoscopy between July 2011 and October 2014. The patients were prospectively recruited in the HELDIVPAT study (ERA-NET consortium), as previously described [26]. Before upper GI endoscopy, information on regular use of PPI, ASA (predominantly LDA) and NSAIDs was collected using a structured questionnaire. Due to the structure of the questionnaire, only information related to the intake and drug class was systematically provided. Information regarding the dose or the art of PPI or NSAIDs was not complete for all subjects, therefore was excluded from subsequent subgroup analysis. Gastric biopsies were obtained from antrum and corpus and were snap-frozen in liquid nitrogen and stored at– 80°C until further use.

### Histological analysis, scoring of immune cell infiltrate and determination of *H*. *pylori*-status

Histological analysis and evaluation of *H*. *pylori* status was performed as previously described [25, 26]. Briefly, gastric mucosa was histologically characterized by standard procedure according to the updated Sydney Classification [28]. The semi-quantitative assessment of polymorphonuclear (PMN) infiltration refers to active inflammation of gastric mucosa and mononuclear (MC) infiltrates to chronicity. Histological assessment was performed as part of the regular standard of care by expert pathologists who were blinded for the treatment or study arm. The scoring based on the Sydney classification was provided to the authors for the further analysis. For activity assessment, the score 0 refers to none PMN infiltration, 1 to mild, 2 to moderate and 3 to severe infiltration with PMNs. For assessment of chronic inflammation, the score 0 refers to none, 1 to mild, 2 to moderate and 3 to severe MC infiltration. *H*. *pylori* infection was evaluated by using histology, urea breath test (^13^C-UBT, only in the first arm of the study), rapid urease test (RUT), by serology and microbiological cultivation. *H*. *pylori*-seropositivity was evaluated by anti-IgG enzyme-linked immunosorbent assay (ELISA) (Pyloriset EIA-GIII, BAG, Lich, Germany) and *Helicobacter pylori* IgG ELISA (Biohit Oyj, Helsinki, Finland) according to the manufacturer’s protocols.

### *In vitro* experiments and Isolation of mucosal CD4+ and CD4- cells

The cultivation experiment of AGS cells was performed as described previously [26]. Three *AGS* cell samples without *H*. *pylori* treatment were used for miR-155 measurement. For *ex vivo* experiments, antrum biopsies were obtained from three patients with chronic non-atrophic gastritis and atrophic gastritis. One of the patients had an active *H*. *pylori gastritis*. All biopsies were digested with collagenase type I (Thermo Fisher Scientific, Waltham, USA) for single cell suspension. The CD4+ T cell positive selection was performed with human CD4 MicroBeads (Miltenyi Biotec GmbH, Bergisch Gladbach, Germany) and separated CD4+ and CD4- cells were stored at -80°C.

### RNA isolation and miRNA quantification in gastric biopsies

Extraction of total RNA (including miRNA) was performed using the Qiagen RNeasyPlus Universal Mini Kit (Qiagen, Hilden, Germany) according to manufacturer’s protocol as previously described [8]. Briefly, the samples were homogenized in QIAzol Lysis Reagent using TissueRuptor. RNA was further precipitated with chloroform, mixed with 1.5 volumes of 100% of Ethanol and following precipitation and washing steps eluted in RNase-free water. The concentration of extracted RNA was assessed using UV-spectrophotometry. MiRNA expression was quantitatively evaluated using either the TaqMan miRNA assay (Applied Biosystems, CA, USA) or SYBR Green (RNU6b) method. Approximately 20 ng of total RNA were reverse transcribed and quantitative real-time PCR analysis was subsequently carried out using the TaqMan Universal PCR Master Mix II (Applied Biosystems) or Power SYBR Green PCR-Master-Mix (Applied Biosystems). MiRNA expression values were subsequently normalized to the small nuclear RNA RNU6b. Following primers were used for the RT-PCR analysis: hsa-miR-155-5p (TaqMan Assay ID: 002623); hsa-miR-223-3p (TaqMan Assay ID: 002295); RNU6b [29].

### Statistical analysis

All data were statistically analyzed using the GraphPad Prism 6.0 software (San Diego, CA, USA). Data are expressed as mean ± SD. To evaluate the statistical difference between the groups a Mann-Whitney U test was used for unpaired, a Wilcoxon test for paired, and Kruskal-Wallis with Dunn´s posttest for multiple variables. A two-sided p-value <0.05 was considered as significant.

## Results

### Influence of *H*. *pylori* on miR-155 and miR-223 expression

The expression of miR-155 and miR-223 was first evaluated with respect to *H*. *pylori*-infection (independently of LDA intake) in an interventional study. As shown in Fig 1B and 1C, the expression pattern of miR-155 and miR-223 was significantly higher in the inflamed gastric mucosa of *H*. *pylori* positive (*H*. *pylori +*) compared to *H*. *pylori* negative (*H*. *pylori -*) subjects (*H*. *pylori+* 0.039±0.017 vs. *H*. *pylori-* 0.024±0.020, p = 0.035 for miR-155; 0.397±0.302 vs. 0.126±0.156, p = 0.0039 for miR-223). Eradication of *H*. *pylori* resulted in a significant improvement of inflammation in antrum and corpus. There was a significant decrease of miR-223 (*H*. *pylori+* 0.397±0.302 vs. *H*. *pylori+/-* 0.073±0.027, p = 0.0078) and a non-significant decrease of miR-155 (0.039±0.017 vs. 0.029±0.007, p = 0.36) already 3 months after eradication (Fig 1B and 1C). Histological assessment confirmed the improvement of inflammation with significantly reduced Sydney scores for activity (p = 0.0039 and p = 0.0156 in antrum and corpus, respectively) and for chronicity (p = 0.0078 in antrum) in subjects after *H*. *pylori* eradication (Fig 1D and 1E).

### Effect of LDA on the expression of miR-155 and miR-223 in gastric mucosa

**Fig 2:**
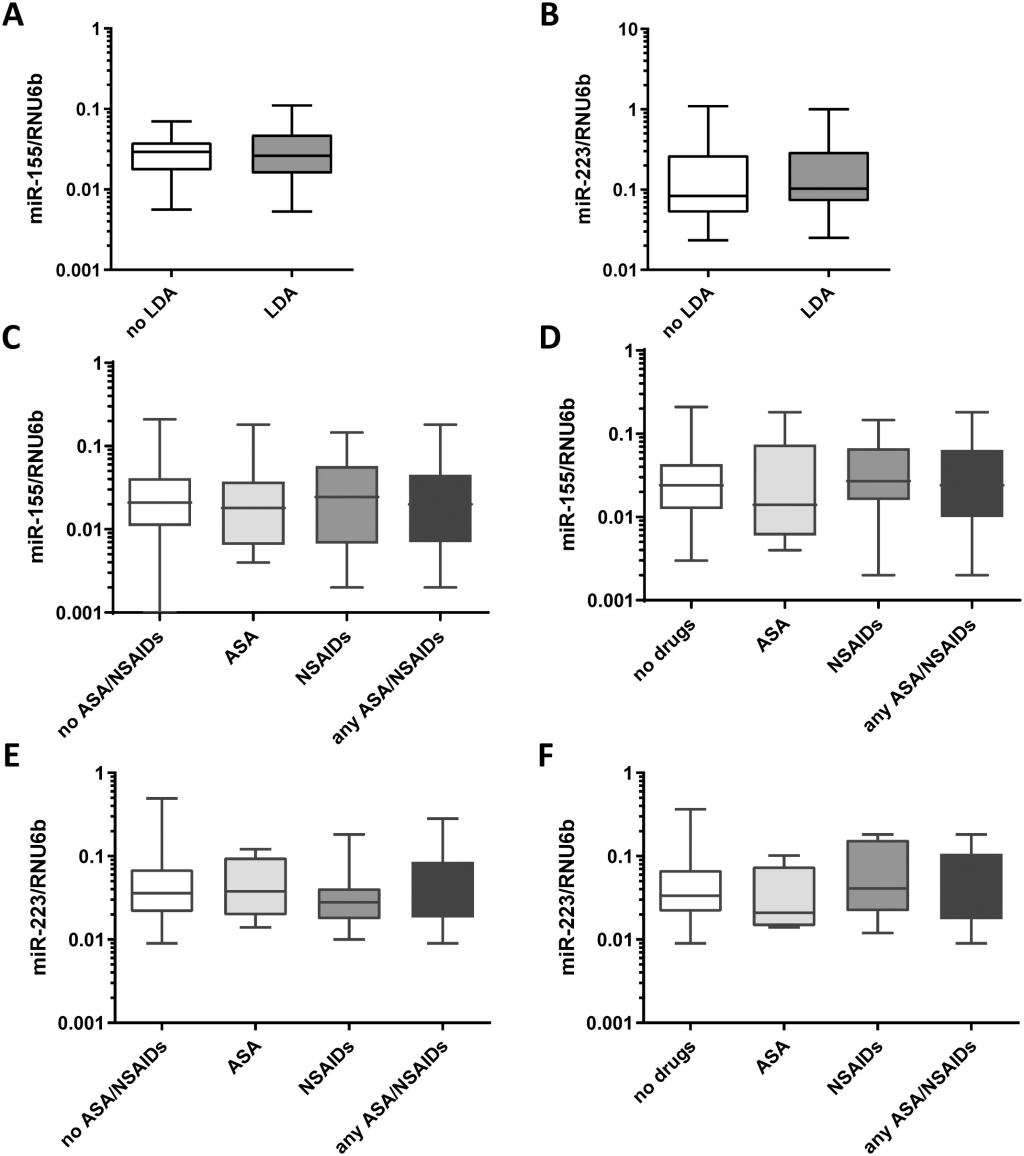
Effect of LDA, ASA or NSAIDs on miR-155 and miR-223 expression. **(A)** MiR-155 and **(B)** miR-223 expression levels in gastric antrum biopsies from healthy subjects of the LDA-study (n = 29) before and after 7 days of daily intake of LDA (100mg). **(C)** Expression of miR-155 in gastric antrum biopsies from patients with no ASA/NSAIDs (n = 113), with ASA (n = 25), NSAIDs (n = 34) or either of drugs (n = 70). **(D)** Expression of miR-155 in mucosa in patients with no PPI intake: no drugs (n = 65), ASA (n = 12), NSAIDs (n = 22) or ASA/NSAIDs (n = 36). **(E)** Expression of miR-223 in gastric antrum biopsies from patients with no ASA/NSAIDs (n = 57), with ASA (n = 13), NSAIDs (n = 17) or either of drugs (n = 36). **(F)** Expression of miR-223 in mucosa in patients with no PPI intake: no drugs (n = 34), ASA (n = 5), NSAIDs (n = 8) or ASA/NSAIDs (n = 17). MiRNA expression levels are shown as 2^ΔCT^-values normalized to RNU6b and data are shown as box-plots where the horizontal line marks the median value.

The effect of LDA intake on the expression of miR-155 and miR-223 was first investigated in healthy volunteers in the interventional study. Study design is shown in the Fig 2. Aim of this explorative study was to evaluate if a short-term intake of LDA could have an effect on inflammatory miRNAs both in *H*. *pylori*-negative and in *H*. *pylori*-positive subjects. After a 7-day LDA exposure, subjects had no significant differences in the level of miR-155 and miR-223 in antrum mucosa compared to subjects without treatment (0.033±0.023 vs. 0.030±0.017, p>0.05, Fig 2A and 2B) respectively. These results were consistently observed independent of the *H*. *pylori*-infection status and time of exposure to LDA (days 0, 1, 3 and 7). Subgroups of *H*. *pylori*-positive subjects, *H*. *pylori*-negative subjects and individuals after *H*. *pylori* eradication showed no differences in miR-155 and miR-223 expressions after a 7-day LDA exposure (S1 Fig).

### Effect of any NSAID on the expression of miR-155 and miR-223 in the gastric mucosa

In the explorative interventional study, a short-term LDA intake did not influence miR-155 and miR-223 expression. To validate our observations and to investigate also the long-term effect of ASA and/or other NSAIDs on these inflammatory miRNAs we evaluated the effect of regular therapy with ASA or other NSAIDs in the larger number of samples from the case-control cohort. This study group included concomitant patients with CNAG±*H*. *pylori* (n = 62), AG±IM (n = 73), peptic ulcer disease (PUD, n = 18), gastric neoplasia (n = 15) and controls (n = 20). Also in these settings, no treatment-related difference in miRNA expression in gastric antral mucosa between ASA/NSAIDs users and non-users were observed (Fig 2). The results were consistent independently to PPI therapy (Fig 2C and 2D).

### Effect of PPI on miR-155 and miR-223 expression

**Fig 3:**
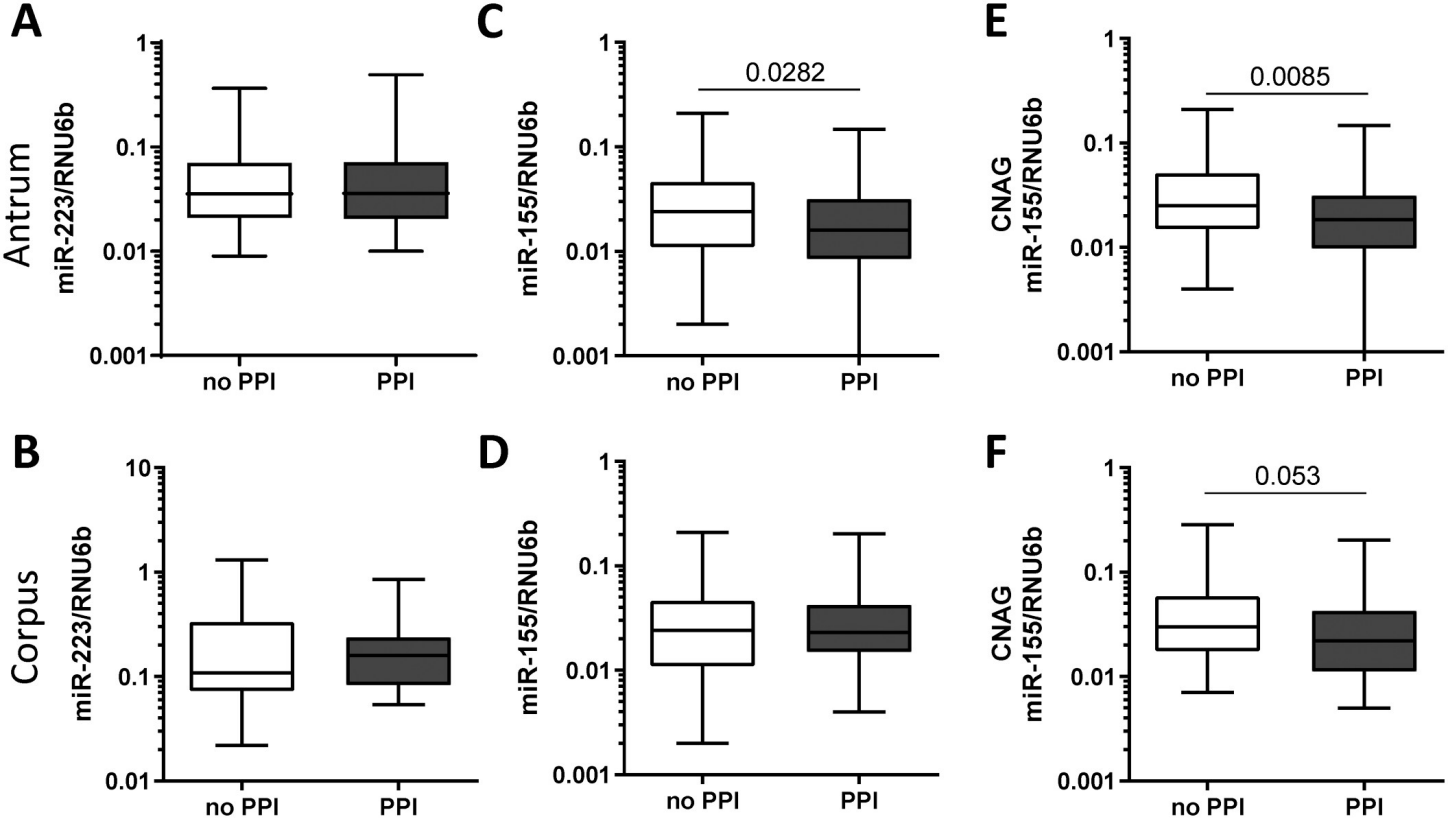
MiR-223 and miR-155 expression in relation to PPI intake in antrum mucosa. MiR-223 expression in gastric biopsies from **(A)** antrum (no PPI n = 52, PPI n = 45) and **(B)** corpus (no PPI n = 34, PPI n = 35) in relation to PPI intake. MiR-155 expression in **(C)** antrum (no PPI n = 103, PPI n = 85) and **(D)** corpus (no PPI n = 103, PPI n = 83). MiR-155 expression in relation to PPI intake in patients with chronic non-atrophic gastritis (CNAG) in **(E)** antrum (no PPI n = 77, PPI n = 58) and **(F)** corpus (no PPI n = 76, PPI n = 56). Values are reported as 2^ΔCT^ -values normalized to RNU6b.

**Fig 4:**
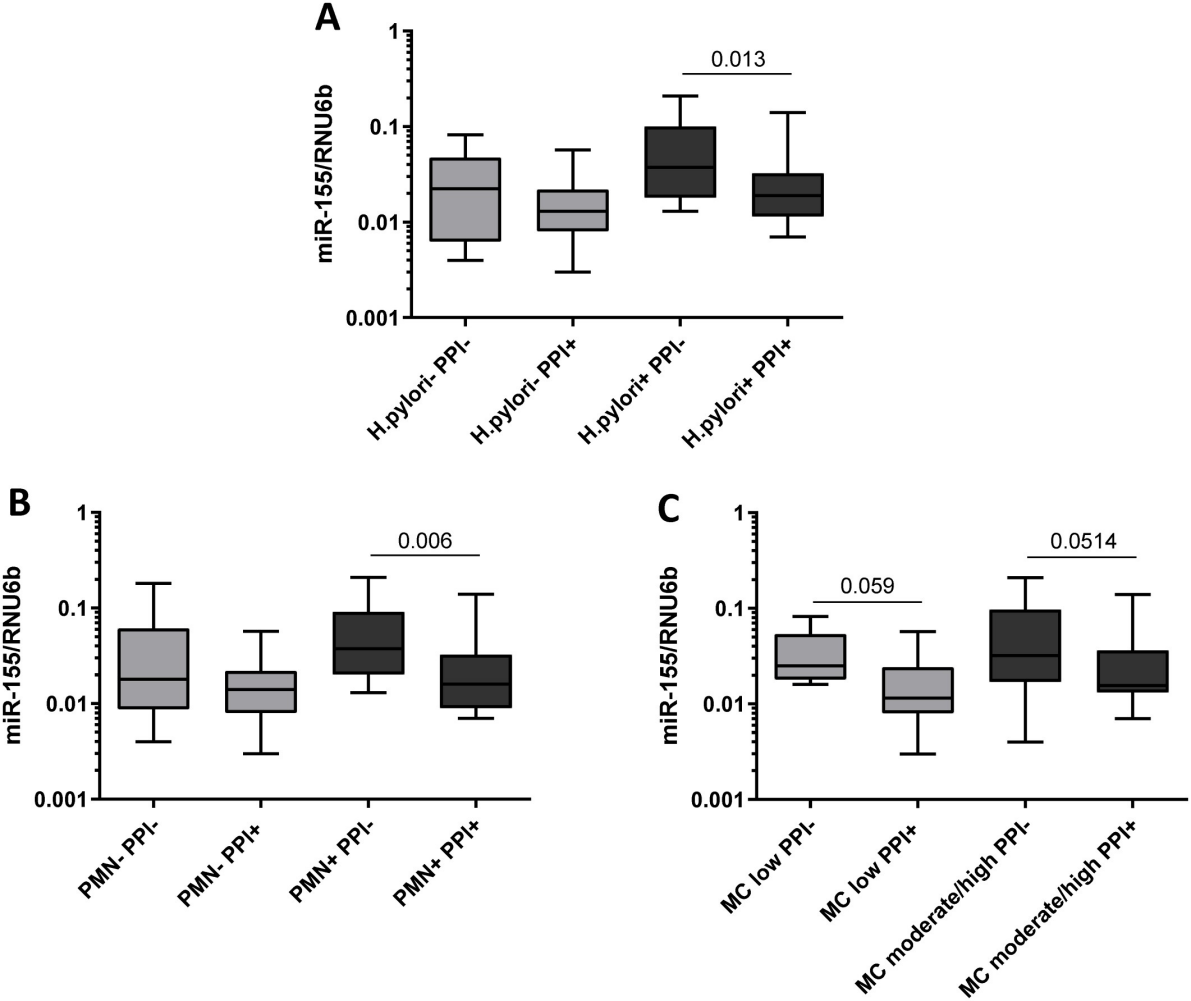
Subgroup analysis of miR-155 expression in regard of *H*. *pylori* infection, activity and chronicity of inflammation. **(A)** Comparison of miR-155 expression in gastric biopsies from antrum between patients with CNAG and with or without regular PPI-intake in relationship to **(A)** *H*. *pylori*-status (*H*. *pylori*-/PPI-, n = 8; *H*. *pylori*-/PPI+, n = 11; *H*. *pylori*+/PPI-, n = 26; *H*. *pylori*+/PPI+, n = 17); (**B**) to the activity of Inflammation based on the updated Sydney score defined by the density of polymorphonuclear (PMN) infiltrate (PMN-/PPI-, n = 10; PMN-/PPI+, n = 11; PMN+/PPI-, n = 24; PMN+/PPI+, n = 17); and (**C**) to chronicity of inflammation based on the updated Sydney score defined by the density of mononuclear infiltrate (MC low PPI-, n = 7; MC low/PPI+, n = 12; MC moderate/high/PPI-, n = 27; MC moderate/high/PPI+, n = 16). PMN- = normal PMN infiltrate; PMN+ = mild-moderate PMN infiltrate; chronicity: MC low = normal/mild mononuclear infiltrate; MC moderate/high = moderate/marked mononuclear infiltrate. Values are reported as 2^ΔCT^-values normalized to RNU6b.

In the case-control study population, we found no significant differences in miR-223 levels between patients exposed to PPI and patients not using PPI both in antrum and in corpus (Fig 3A and 3B). In comparison, patients under the PPI therapy showed significantly lower miR-155 expression in antrum (no PPI 0.038±0.040 vs. PPI 0.027±0.031, p = 0.0282, Fig 3C), whereas no changes of miR-155 expression were observed in corpus mucosa (0.038±0.040 vs. 0.033±0.028, p = 0.683, Fig 3D). Since the influence of PPI may differ dependent on the state of inflammation, we performed a subgroup analysis on how PPI-intake was related to miR-155 expression according to the histological status. Only patients with CNAG (under PPI-therapy, n = 58; no PPI-therapy, n = 77) showed reduced miR-155 level in antrum (0.041±0.041 vs. 0.028±0.032, p = 0.0085, Fig 3E) but not patients with other conditions (AG, IM, PUD) or controls with normal mucosa. A similar trend was observed in corpus mucosa of patients with CNAG, but it did not reach the statistical significance (0.045±0.045 vs. 0.032±0.032, p = 0.053, Fig 3F). Specifically, among the patients with CNAG the decrease in miR-155 expression was observed predominantly in subjects with *H*. *pylori*-infection (*H*. *pylori*+/PPI- 0.060±0.054 vs. *H*. *pylori*+/PPI+ 0.030±0.034, p = 0.013, Fig 4A). To analyze whether the decreased expression of miR-155 observed in patients taking PPI was related to the degree of inflammatory cells infiltrating the gastric mucosa, we compared samples with the similar grade of neutrophils or monocytes infiltrate. As shown in Fig 4, miR-155 expression was lower in biopsies with high levels of polymorphonuclear infiltration (PMN+) from patients exposed to PPI treatment compared to biopsies with the same degree of infiltration from patients without PPI therapy (PMN+/PPI- 0.058±0.050 vs. PMN+/PPI+ 0.029±0.034, p = 0.006). Minimal non-significant changes were observed in patients with normal density of intraepithelial neutrophils (PMN-/PPI- 0.042±0.055 vs. PMN-/PPI+ 0.017±0.015, p>0.05). The association between PPI intake and reduced miR-155 expression was also observed by comparing samples with the same level of mononuclear cells infiltration (MC), a marker of chronic inflammation. MiR-155 expression was, indeed, slightly lower in gastric mucosa of patients using PPI in both subgroups with low (MC low/PPI- 0.038±0.025 vs. MC low/PPI+ 0.017±0.015, p = 0.059) and moderate/high mononuclear infiltrate (MC moderate/high/PPI- 0.057±0.055 vs. MC moderate/high/PPI+ 0.030±0.035, p = 0.0514, Fig 4C).

### Expression in miR-155 in AGS cells and mucosal CD4+/- cells

It is known that gastric mucosa shows only low to moderate miR-155 expression and it is hypothesized that the increased expression of miR-155 in mucosa may be related to the infiltrating immune cells including lymphocytes, which show very high level of miR-155 expression. In order to evaluate the miR-155 expression in gastric mucosa, we cultivated gastric cancer AGS cells and isolated mucosal CD4+ immune cells from antral gastric biopsies of patients with chronic gastritis. AGS cells without *H*. *pylori* treatment showed very low but detectable miR-155 expression compared to CD4- and CD4+ cells from gastric mucosa of *H*. *pylori* infected (n = 1) and non-infected patients (n = 2) (S2 Fig). Those data suggest that alteration in miR-155 expression, for instance following PPI treatment, may be predominantly related to the changes in inflammatory cells infiltrating the gastric mucosa as the miR-155 changes in epithelial cells.

## Discussion

There is limited knowledge regarding the potential influence of commonly used drugs such as anti-inflammatory drugs or PPI on mucosal miRNAs. In this study, we evaluated the effect of *H*. *pylori* eradication, LDA, NSAIDs and PPI on the expression of two miRNAs that are believed to play an important role in the regulation of inflammatory processes and are deregulated in preneoplastic conditions of the gastric mucosa [8–13]. Using two different cohorts (a prospective arm with healthy subjects and a case-control study), we demonstrate that ASA and NSAIDs did not influence miRNA expression, while PPI treatment was associated with reduced expression of miR-155, particularly in *H*. *pylori*-positive subjects without AG.

We detected high levels of both miR-223 and miR-155 in the gastric mucosa form *H*. *pylori*-infected otherwise healthy volunteers. This is in agreement with previous evidence, showing that miR-155 and miR-223 are frequently deregulated both in corpus and antrum of *H*. *pylori*-positive subjects and strongly associated with gastric preneoplastic conditions such as AG and IM [8, 11]. *H*. *pylori* eradication led to a significant reduction of miR-223 and a trend for reduction of miR-155, which levels may remain still elevated on the short term (3 months after therapy) due to a possible longer-lasting immune memory. The observed changes correlated very well with the improvement of the Sydney-scores for active and chronic inflammation in histopathological assessment, indicating also that both miR-155 and miR-223 expressions predominantly originate from immune cells infiltrating the gastric mucosa and are reduced when the immune infiltrate declines after eradication. Our *in-vitro* experiments, support the origin of inflammatory miRNAs. In fact, we showed that the main source of miR-155 may be immune cells (CD4+/-), while the gastric cell line AGS used for comparison showed only very low expression of the studied inflammatory miRNA. Accordingly, previous studies aiming to characterize the cell-type specific miRNAs profile and their regulatory role at epigenetic level reported that miR-223 was specific to myeloid lineage cells (neutrophils, eosinophils and monocytes), whereas miR-155 was specific to lymphoid lineage cells (plasmacytoid dendritic cells, T cells, B cells and NK cells) [30]. In the context of a *H*. *pylori*-infection the expression of miR-223, that plays a critical role in development and regulation of granulocytes [31], is elevated and directly linked to the degree of neutrophils infiltrate within the gastric mucosa [11]. The induction of miR-155 upon *H*. *pylori*-infection has been also previously reported [9, 10, 32]. MiR-155 is present at low levels in epithelial tissue under normal physiological conditions and has been recognized as a crucial regulator of the immune response to *H*. *pylori* promoting Th1 and Th17 differentiation, which is necessary to control the infection but related to a major risk of developing preneoplastic gastric entities [10, 33].

It is known that ASA and NSAIDs may induce gastritis and lead to PUD. NSAIDs have been shown to affect the gene expression through alteration of various transcription factors and modulation of different epigenetic mechanisms, including influence on different miRNAs expression [34]. According to our data, the level of miR-155 and miR-223 expression was similar during the course of LDA treatment and was at least partially dependent on the presence of *H*. *pylori* infection, as previously hypothesized. Although the most common effects of ASA are expected to be detectable already in the short-term period, it is potentially possible that this time frame might be too short. To evaluate whether a regularly and prolonged use of ASA may influence the expression of inflammatory miRNA in gastric mucosa we performed a validation analysis in a second cohort that not only included patients treated with ASA (including LDA) bus also with NSAIDs. The data of this prospective analysis in similar fashion showed negative results in agreement with those of the first explorative study. Although both studies provide concordant evidence, the conclusions may still be limited to the two selected inflammatory miRNAs and do not consider a brighter miRNA-profile.

PPIs are frequently prescribed because of epigastric pain or dyspepsia, however, the impact of PPI on mucosal miRNA has not been studied yet. According to our results, intake of PPI is associated with a significant reduction of miR-155 but not miR-223 expression, which is more evident in *H*. *pylori*-infected mucosa and in the stomach antrum. It has been shown that inhibition of acid secretion following long-term PPI intake alters *H*. *pylori*-related gastritis reducing the total number of bacteria colonizing the stomach, and modifying the inflammatory pattern from being antral-predominant to being body-predominant, with a higher intensity of inflammation within the acid-secreting mucosa [35]. Thus, one might argue that the decreased levels of miR-155 observed in antrum in PPI+ subject may be a consequence of the effect of acid-suppression on *H*. *pylori*-density and secondary to the inflammation in antrum mucosa. However, this effect seems to be at least partially independent from the degree of active (PMNs) and/or chronic inflammation (MCs) of the gastric mucosa. Indeed, significantly lower levels of miR-155 were detected in patients with PPI intake and matching density of PMNs and MCs. The reduction of inflammatory miRNA expression observed in this work may be one of the additional mechanisms.

Recently, an increasing number of observational studies linked PPI intake with an increased risk (up to 2fold) of gastric cancer [36, 37]. Nevertheless, the association between long-term PPI use and increased risk of GC remains controversial and high-quality prospective studies are urgently needed. Nevertheless, at present it is unclear if potential inadequate adjustment for confounders including persistence of *H*. *pylori* or additional factors including (smoking, obesity and alcohol) could at least partially contributed to the results. Most importantly, an increased incidence may be attributed to the *H*. *pylori* persistence or to precancerous conditions such as (AG or IM) that could be present at baseline of the study. The relationship between acid-suppression, inflammation and carcinogenesis needs to be further in depth assessment in future studies.

This study was the first to analyze in humans the effects of ASA, NSAIDs and PPI on inflammatory miRNA expression in gastric mucosa. We believe this analysis on a cohort of healthy volunteers with or without *H*. *pylori* infection challenged with LDA and the validation on an independent cohort of prospectively recruited patients provides the highest level of evidence. Nevertheless, some limitations have to be noted. Firstly, we limited the analysis to miR-155 and miR-223, that are to our view the most important miRNAs involved in *H*. *pylori*-related inflammation. Other miRNAs may still be influenced by long-term intake of ASA/NSAIDs or PPI. Secondly, the scoring of immune cell infiltrate relied on a semi-quantitative histological evaluation of the gastric mucosa based on the updated Sydney-classification, which represents the actual standard of care in clinical setting. An in-depth analysis of the immune cell infiltration that allows the differentiation of mononuclear cells and the identification of activated immune cells would certainly provide more detailed information of the immune response and should be performed in future studies addressing the role of miRNAs in the inflammatory process of the gastric mucosa. Furthermore, we recorded information regarding the NSAIDs, ASA, PPI intake, but no systematic data was available to determine the dose effect and possible correlation to specific drug types.

In conclusion, our data provide additional evidence supporting the potential value of miRNAs in *H*. *pylori*-related pathology and gastric inflammation. PPI but not NSAIDs or low-dose aspirin are associated with changes in expression of mucosal inflammatory miRNAs in *H*. *pylori*-dependent manner. Further studies are needed to elucidate the potential clinical implication of PPI-related miR-155 modification of carcinogenesis in *H*. *pylori*-related diseases.

## Supporting information

S1 Fig Effect of LDA on miR-155 and miR-223 expression in gastric mucosa in subgroups dependent on *H*. *pylori* status. Time- and *H*. *pylori*-dependent patterns of (A-C) miR-155 and (D-F) miR-223 expression in gastric antral mucosa of healthy subjects (n = 29). (A and D) show the *H*. *pylori* negative subjects, (B and E) show the results in *H*. *pylori* positive subjects. (C and F) show the results of 9 subjects from *H*. *pylori* positive group 3 months after *H*. *pylori* eradication. MiRNA expression levels are shown as 2^ΔdCT^-values normalized to RNU6b and data are shown as box-plots where the horizontal line marks the median value.
(TIF)

S2 Fig MiR-155 expression in gastric cancer cell lines and CD4+/- cell ex vivo. **(A)** Normalized miR-155 expression and **(B)** raw miR-155 CT values of CD4+ and CD4- cells from *H*. *pylori* negative (n = 2) and *H*. *pylori* positive (n = 1) patients and AGS cells (n = 3) without *H*. *pylori* treatment. Normalized values are reported as 2^ΔCT^-values normalized to RNU6b.
(TIF)

S1 Data (PDF)
