# Supplementary figures and images for: Inflammatory microRNAs in gastric mucosa are modulated by Helicobacter pylori infection and proton-pump inhibitors but not by aspirin or NSAIDs
Source: PLoS One. 2021 Apr 15;16(4):e0249282. doi: 10.1371/journal.pone.0249282 (PMC8049315; doi:10.1371/journal.pone.0249282)

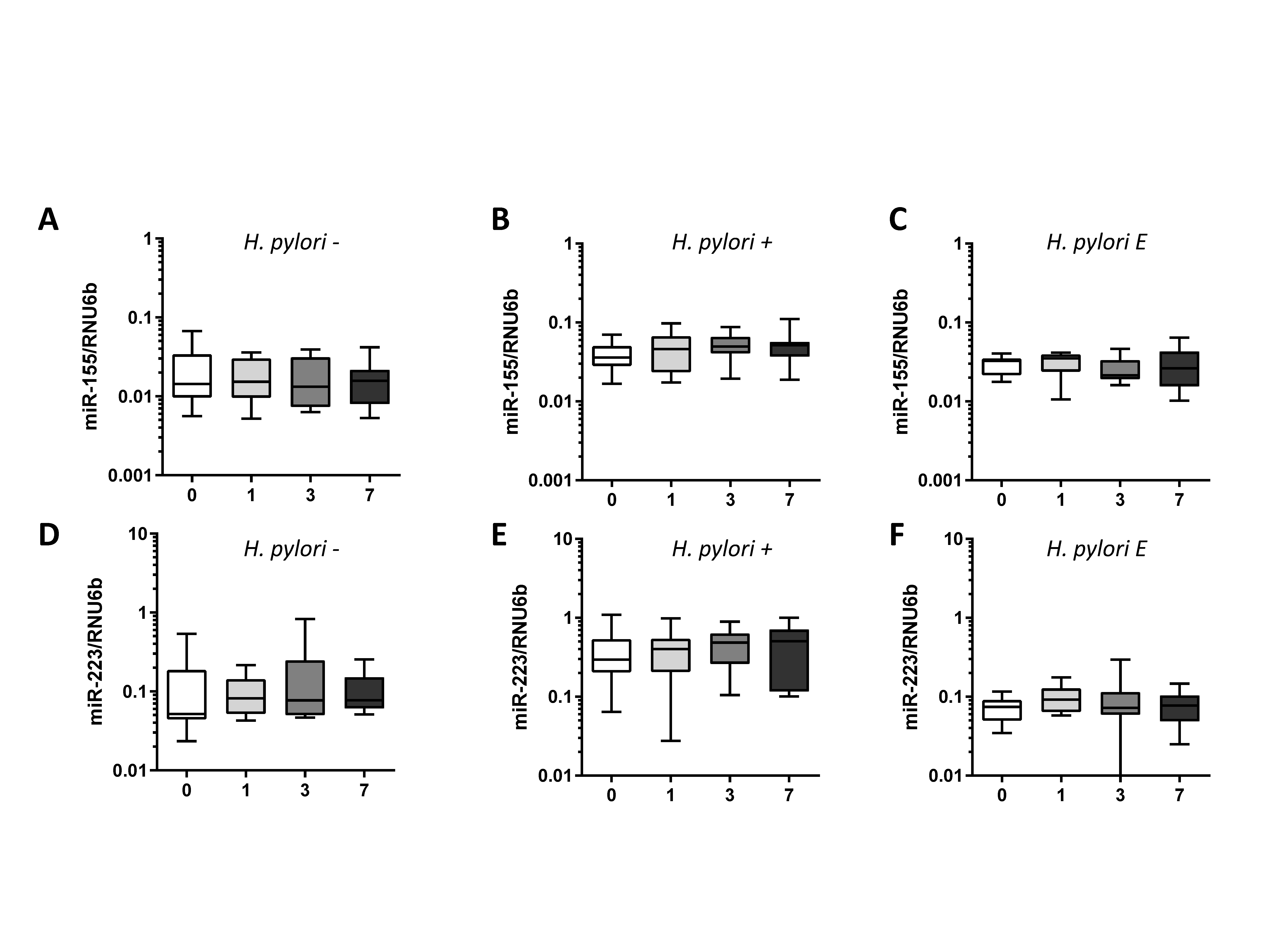

Supplement: S1 Fig — Time- and H. pylori-dependent patterns of (A-C) miR-155 and (D-F) miR-223 expression in gastric antral mucosa of healthy subjects (n = 29). (A and D) show the H. pylori negative subjects, (B and E) show the results in H. pylori positive subjects. (C and F) show the results of 9 subjects from H. pylori positive group 3 months after H. pylori eradication. MiRNA expression levels are shown as 2ΔdCT-values normalized to RNU6b and data are shown as box-plots where the horizontal line marks the median value. (TIF) [file pone.0249282.s001.tif]
