# Supplementary material for: Inflammatory microRNAs in gastric mucosa are modulated by Helicobacter pylori infection and proton-pump inhibitors but not by aspirin or NSAIDs
Source: PLoS One. 2021 Apr 15;16(4):e0249282. doi: 10.1371/journal.pone.0249282 (PMC8049315; doi:10.1371/journal.pone.0249282)
Supplement: S1 Data — (PDF) [file pone.0249282.s003.pdf]

Nr.	Tag	Gruppe	miR155	miR223
a	0	0	0,0124	0,0451
a	1	0	0,0153	0,0754
a	3	0	0,0085	0,0575
a	7	0	0,0066	0,0511
b	0	1	0,0703	0,2994
b	1	1	0,0587	0,4475
b	3	1	0,0616	0,6071
b	7	1	0,0556	1,0000
b	0	E	0,0250	0,0356
b	7	E	0,0448	0,0448
b	1	E	0,0404	0,0579
b	3	E	0,0464	0,1088
c	1	1	0,0494	0,9862
c	3	1	0,0464	0,5249
c	0	1	0,0167	0,0643
c	7	1	0,1103	0,4698
c	0	E	0,0335	0,0743
c	7	E	0,0407	0,1029
c	1	E	0,0374	0,0608
c	3	E	0,0315	0,0723
d	1	0	0,0274	0,1684
d	3	0	0,0069	0,0504
d	0	0	0,0294	0,1843
d	7	0	0,0167	0,1792
e	1	1	0,0974	0,2793
e	3	1	0,0194	0,3276
e	0	1	0,0292	0,2253
e	7	1	0,0556	0,1174
e	0	E	0,0177	0,0347
e	7	E	0,0138	0,0250
e	1	E	0,0186	0,0718
e	3	E	0,0161	0,0567
f	1	0	0,0310	0,0813
f	3	0	0,0326	0,8236
f	0	0	0,0098	0,0404
f	7	0	0,0169	0,1416
g	0	0	0,0163	0,0470
g	7	0	0,0105	0,0902
h	1	0	0,0292	0,2161
h	3	0	0,0393	0,2952
h	0	0	0,0474	0,5359
h	7	0	0,0237	0,0791
i	1	0	0,0110	0,0442
i	3	0	0,0272	0,1044
i	0	0	0,0272	0,1961
i	7	0	0,0209	0,2553
j	1	0	0,0081	0,0591
j	3	0	0,0141	0,0967
j	0	0	0,0091	0,0552
j	7	0	0,0084	0,0625
k	1	1	0,0190	0,2222
k	3	1	0,0780	0,4444
k	0	1	0,0652	1,0943
k	7	1	0,0470	0,3322
k	0	E	0,0219	0,1166
k	7	E	0,0354	0,1044
k	1	E	0,0106	0,0670
k	3	E	0,0195	0,1207
l	1	0	0,0359	0,1073
l	0	0	0,0670	0,0836
l	7	0	0,0418	0,0759
m	1	0	0,0052	0,0427
m	3	0	0,0063	0,0467
m	0	0	0,0056	0,0234
m	7	0	0,0053	0,0556
n	1	0	0,0141	0,1166
n	3	0	0,0123	0,0497
n	0	0	0,0110	0,0484
n	7	0	0,0147	0,0759
o	1	1	0,0249	0,1582
o	3	1	0,0412	0,1719
o	0	1	0,0282	0,2449
o	7	1	0,0188	0,1008
o	0	E	0,0354	0,0689
o	7	E	0,0227	0,0526
o	1	E	0,0319	0,1340
o	3	E	0,0187	0,0643
p	1	1	0,0173	0,0276
p	3	1	0,0511	0,1051
p	0	1	0,0342	0,1406
p	7	1	0,0490	0,1142
p	0	E	0,0326	0,0638
p	7	E	0,0102	0,0981
p	1	E	0,0352	0,1216
p	3	E	0,0249	0,0616
q	1	1	0,0915	0,6598
q	3	1	0,0872	0,7071
q	0	1	0,0454	0,6736
q	7	1	0,0288	0,8827
q	0	E	0,0209	0,0813
q	7	E	0,0172	0,0775
q	1	E	0,0290	0,0921
q	3	E	0,0215	0,0086
r	1	1	0,0544	0,4569
r	3	1	0,0387	0,2912
r	0	1	0,0379	0,4897
r	7	1	0,0604	0,6507
s	1	1	0,0421	0,5035
s	3	1	0,0604	0,8950
s	0	1	0,0390	0,4475
s	7	1	0,0537	0,5396
s	0	E	0,0347	0,0791
s	7	E	0,0643	0,1466
s	1	E	0,0385	0,1768
s	3	E	0,0354	0,2952
t	1	1	0,0430	0,3536
t	3	1	0,0484	0,5396
t	0	1	0,0280	0,2912
t	7	1	0,0398	0,5396
t	0	E	0,0404	0,1001
t	1	E	0,0415	0,1058
t	3	E	0,0212	0,1022
t	7	E	0,0263	0,0675

	Fig 2c				Fig 2d				Fig 2e				Fig 2f		
o ASA/NSAIL	ASA	NSAIDs	any ASA/NSAIDs	no drugs	ASA	NSAIDs	ny ASA/NSAII	o ASA/NSAIL	ASA	NSAIDs	ny ASA/NSAII	no drugs	ASA	NSAIDs	ny ASA/NSAIDs
0,017	0,008	0,024	0,024	0,017	0,181	0,024	0,024	0,047	0,038	0,045	0,045	0,047		0,045	0,045
0,012	0,181	0,064	0,064	0,067	0,006	0,01	0,01						0,048	0,037	0,037
0,022	0,006	0,01	0,01	0,019	0,004	0,044	0,044			0,037	0,037	0,028	0,021		
0,067	0,004	0,031	0,031	0,009	0,01	0,146	0,146		0,048						
0,019	0,01	0,006	0,006	0,041	0,006	0,002	0,181	0,028	0,021				0,015	0,153	
0,009	0,025	0,044	0,044	0,009	0,111	0,1	0,002				0,283	0,013		0,028	0,009
0,041	0,006	0,146	0,044	0,025	0,02	0,03	0,1								0,153
0,009	0,02	0,1	0,146	0,015	0,018	0,083	0,006	0,013	0,015	0,153		0,033		0,159	0,048
0,096	0,006	0,03	0,008	0,009	0,043	0,029	0,004		0,091	0,028	0,038	0,018	0,102	0,183	0,021
0,011	0,078	0,003	0,181	0,014	0,084	0,018	0,01	0,073		0,012		0,063			
0,025	0,014	0,002	0,002	0,012	0,007	0,067	0,03		0,038	0,01	0,009	0,032	0,014	0,012	0,028
0,05	0,009	0,006	0,1	0,021	0,004	0,055	0,083			0,034	0,153	0,105		0,02	
0,015	0,032	0,083	0,02	0,005		0,003	0,006	0,033				0,022			0,015
0,009	0,111	0,029	0,006	0,014		0,01	0,029	0,018		0,159	0,048	0,132			0,159
0,014	0,005	0,02	0,004	0,003		0,067	0,018	0,063	0,069	0,035	0,021	0,021			0,055
0,012	0,097	0,048	0,01	0,037		0,104	0,042	0,032	0,117						
0,021	0,02	0,006	0,03	0,027		0,018	0,067	0,105		0,017	0,028				0,183
0,005	0,022	0,067	0,003	0,041		0,005	0,055	0,022	0,028	0,183	0,012				
0,023	0,018	0,055	0,025	0,018		0,052	0,003		0,018						0,012
0,014	0,043	0,003	0,002	0,033		0,024	0,01	0,132		0,012	0,01	0,034			0,02
0,003	0,084	0,01	0,006	0,151		0,025	0,067	0,021		0,02	0,034	0,245			
0,037	0,007	0,015	0,083	0,015		0,024	0,104		0,121	0,035		0,054			
0,027	0,02	0,008	0,006	0,066			0,111			0,023	0,015				
0,041	0,007	0,14	0,029	0,024			0,018		0,102		0,159	0,045			0,112
0,047	0,004	0,104	0,018	0,017			0,019				0,055	0,069			0,028
0,01		0,003	0,02	0,029			0,02	0,038	0,014	0,024	0,091				
0,018		0,127	0,02	0,133			0,005				0,035				
0,147		0,005	0,048	0,054			0,018	0,493							
0,008		0,007	0,042	0,003			0,043	0,015		0,018		0,009			0,102
0,033		0,052	0,006	0,004			0,084	0,034			0,038	0,021			
0,026		0,024	0,006	0,015			0,052	0,108			0,017	0,027			
0,066		0,025	0,023	0,097			0,024				0,037				
0,01		0,024	0,067	0,082			0,007	0,029			0,183				0,014
0,151		0,025	0,055	0,013			0,025	0,245				0,024			
0,022			0,003	0,073			0,024				0,012				
0,002			0,01	0,12			0,004	0,015			0,02				
0,018			0,015	0,112				0,07			0,035				
0,015			0,005	0,025				0,054			0,043	0,127			
0			0,016	0,057				0,109				0,163			
0,024			0,078	0,026				0,073				0,038			
0,066			0,067	0,005								0,013			
0,013			0,014	0,038											
0,024			0,008	0,011				0,045			0,023				
0,017			0,009	0,043				0,069			0,069				
0,029			0,14	0,046								0,063			
0,014			0,104	0,028				0,045				0,107			
0,014			0,032	0,022				0,036			0,117	0,02			
0,005			0,111	0,046				0,034				0,366			
0,133			0,003	0,21							0,024				
0,019			0,018	0,05				0,054			0,112				
0,054			0,019	0,006							0,028	0,01			
0,003			0,005	0,006				0,009			0,018	0,049			
0,004			0,097	0,03				0,021							
0,013			0,02	0,01				0,046							
0,057			0,127	0,014											
0,015			0,022	0,032				0,027			0,121				
0,097			0,005	0,022								0,033			
0,009			0,007	0,043				0,293			0,018	0,068			
0,082			0,018	0,004											
0,013			0,043	0,016				0,024			0,102				
0,073			0,084	0,008								0,028			
0,016			0,052	0,02				0,066				0,03			
0,12			0,024	0,037											
0,112			0,007	0,025							0,014				
0,025			0,025	0,011				0,127							
0,014			0,024												
0,057			0,02					0,163							
0,026			0,007					0,038							
0,005			0,004					0,013							
0,038			0,025												
0,008															
0,011															
0,003								0,013							
0,043															
0,046								0,063							
0,028								0,107							
0,022								0,02							
0,046								0,366							
0,109															
0,21															
0,026															
0,038															
0,044															
0,05															
0,025															
0,04															
0,006								0,01							
0,021															
0,006								0,049							
0,01								0,024							
0,03															
0,01															
0,014															
0,032															
0,022								0,033							
0,043								0,068							
0,01								0,014							
0,004															
0,016															
0,008								0,028							
0,02								0,03							
0,002								0,024							
0,014															
0,037															
0,025															
0,015															
0,009								0,013							
0,01															
0,013								0,016							
0,024															
0,011															
0,048															
0,054															

Fig 3a		Fig 3b		Fig 3c		Fig 3d		Fig 3e		Fig 3f	
no PPI	PPI	no PPI	PPI	no PPI	PPI	no PPI	PPI	no PPI	PPI	no PPI	PPI
0,045		0,092		0,024	0,064	0,024	0,026	0,024	0,012	0,035	0,011
0,047				0,017	0,012	0,017	0,011	0,017	0,022	0,01	0,014
0,037				0,01	0,022	0,01	0,014	0,01	0,031	0,021	0,011
				0,067	0,031	0,067	0,011	0,067	0,006	0,033	0,007
0,028		0,022		0,019	0,006	0,019	0,007	0,009	0,044	0,007	0,077
	0,283			0,009	0,044	0,009	0,077	0,041	0,096	0,019	0,071
				0,041	0,096	0,041	0,071	0,181	0,008	0,11	0,04
0,013	0,073	0,036	0,847	0,009	0,011	0,009	0,04	0,025	0,023	0,02	0,021
	0,038		0,172	0,044	0,008	0,044	0,04	0,015	0,047	0,053	0,021
				0,146	0,05	0,146	0,071	0,1	0,025	0,049	0,046
				0,181	0,02	0,181	0,043	0,014	0,147	0,036	0,203
				0,025	0,023	0,025	0,021	0,021	0,008	0,021	0,043
0,033	0,012	0,192	0,109	0,015	0,003	0,015	0,02	0,006	0,026	0,083	0,083
0,018		0,025		0,009	0,047	0,009	0,021	0,014	0,02	0,106	0,02
0,009	0,038	0,093	0,47	0,002	0,01	0,002	0,024	0,01	0,02	0,013	0,022
0,153				0,1	0,025	0,1	0,046	0,03	0,01	0,019	0,036
0,063	0,01	0,266	0,054	0,014	0,002	0,014	0,028	0,037	0,022	0,022	0,011
0,032	0,493	0,024		0,012	0,147	0,012	0,203	0,027	0,018	0,029	0,04
0,105	0,034		0,068	0,021	0,006	0,021	0,022	0,041	0	0,012	0,02
0,048	0,015	0,582	0,085	0,006	0,008	0,006	0,043	0,018	0,015		0,082
0,022	0,032	0,024	0,299	0,005	0,013	0,005	0,058	0,029	0,005	0,072	0,033
0,132	0,108	0,908	0,199	0,014	0,026	0,014	0,083	0,033	0,078	0,073	0,039
0,021	0,091	0,088	0,245	0,004	0,02	0,004	0,02	0,018	0,014	0,056	0,031
	0,035		0,219	0,01	0,02	0,01	0,022	0,042	0,008	0,066	0,03
0,028				0,03	0,066	0,03	0,064	0,151	0,009	0,221	0,033
0,021		0,127		0,003	0,048	0,003	0,019	0,067	0,14	0,019	0,098
	0,027		0,113	0,037	0,003	0,037	0,027	0,055	0,014	0,011	0,029
	0,029		0,168	0,027	0,01	0,027	0,036	0,015	0,032	0,057	0,045
	0,038		0,159	0,041	0,006	0,041	0,014	0,01	0,019	0,08	0,047
				0,018	0,022	0,018	0,011	0,066	0,013	0,028	0,023
	0,015		0,058	0,083	0,002	0,083	0,015	0,024	0,057	0,071	0,037
0,015	0,017	0,047	0,083	0,006	0,006	0,006	0,023	0,017	0,009	0,03	0,022
0,159	0,07	0,371	0,156	0,029	0,018	0,029	0,04	0,029	0,016	0,039	0,06
0,034	0,037		0,237	0,033	0,023	0,033	0,042	0,104	0,127	0,059	0,046
0,055	0,109	0,257		0,018	0	0,018	0,02	0,133	0,014	0,036	0,009
	0,035		0,432	0,042	0,015	0,042	0,082	0,111	0,022	0,119	0,008
0,245	0,073		0,182	0,151	0,024	0,151	0,025	0,018	0,008	0,04	0,019
0,183				0,067	0,013	0,067	0,011	0,019	0,007	0,049	0,025
	0,043		0,182	0,055	0,005	0,055	0,033	0,054	0,003	0,036	0,017
0,054		0,212		0,015	0,016	0,015	0,015	0,015	0,045	0,047	0,046
0,012		0,075		0,003	0,078	0,003	0,039	0,097	0,026	0,047	
0,02	0,045	0,374	0,066	0,01	0,014	0,01	0,031	0,082	0,038	0,077	0,045
				0,066	0,014	0,066	0,028	0,013	0,044	0,038	0,022
0,045	0,023	0,066	0,074	0,024	0,008	0,024	0,03	0,073	0,025	0,03	0,02
0,069	0,069	0,092	0,247	0,017	0,009	0,017	0,033	0,12	0,04	0,093	0,011
				0,029	0,14	0,029	0,098	0,112	0,01	0,093	0,009
	0,036		0,061	0,067	0,014	0,067	0,029	0,025	0,01	0,027	0,012
	0,034		0,227	0,104	0,005	0,104	0,034	0,057	0,02	0,285	0,015
	0,117			0,133	0,032	0,133	0,045	0,026	0,002	0,013	0,009
	0,054		0,678	0,111	0,019	0,111	0,047	0,005	0,014	0,033	0,011
	0,024		0,106	0,028	0,003	0,028	0,014	0,038	0,015	0,014	0,009
0,112	0,018	0,198	0,095	0,018	0,005	0,018	0,035	0,011	0,009	0,009	0,01
0,028	0,046	0,325	0,091	0,019	0,013	0,019	0,023	0,018	0,01	0,011	0,016
				0,054	0,057	0,054	0,037	0,043	0,013	0,084	0,011
0,009	0,293	0,093	0,221	0,003	0,009	0,003	0,022	0,046	0,007	0,038	0,005
0,021		0,096		0,004	0,097	0,004	0,092	0,028	0,024	0,066	0,017
0,027	0,066	0,121	0,444	0,015	0,016	0,015	0,06	0,022	0,025	0,025	0,019
				0,097	0,127	0,097	0,046	0,046	0,048	0,076	
				0,082	0,014	0,082	0,009	0,21		0,114	
0,024	0,121	0,082	0,093	0,013	0,022	0,013	0,008	0,052		0,012	
				0,073	0,008	0,073	0,019	0,05		0,021	
	0,018		0,076	0,02	0,007	0,02	0,025	0,024		0,013	
	0,013		0,066	0,12	0,003	0,12	0,017	0,007		0,009	
	0,101			0,112	0,045	0,112	0,046	0,006		0,015	
0,127		0,412		0,025	0,109	0,025	0,068	0,01		0,008	
0,163		1,31		0,057	0,026	0,057		0,025		0,017	
0,038				0,026	0,038	0,026	0,045	0,032		0,028	
0,013		0,051		0,005	0,044	0,005	0,022	0,022		0,021	
				0,005	0,025	0,005	0,02	0,043		0,031	
				0,038	0,04	0,038	0,011	0,024		0,027	
				0,011	0,021	0,011	0,004	0,004		0,013	
0,071	0,024	0,171		0,01	0,01	0,01	0,009	0,016		0,026	
	0,014			0,018	0,01	0,018	0,012	0,008		0,021	
0,102				0,043	0,02	0,043	0,015	0,02		0,008	
	0,024			0,043	0,002	0,043	0,009	0,037		0,017	
				0,084	0,014	0,084	0,011	0,004		0,012	
0,063		0,351		0,046	0,015	0,046	0,009	0,011		0,029	
0,107	0,013	0,089		0,028	0,009	0,028	0,01				
0,02				0,022	0,01	0,022	0,016				
0,366	0,016	0,444		0,046	0,013	0,046	0,011				
				0,21	0,007	0,21	0,005				
				0,052	0,024	0,052	0,017				
				0,05	0,025	0,05	0,019				
				0,024	0,048	0,024					
0,014				0,007	0,054	0,007	0,021				
0,01				0,006		0,006					
0,049				0,006		0,006					
				0,03		0,03					
				0,01		0,01					
				0,014		0,014					
				0,025		0,025					
				0,032		0,032					
0,033				0,022		0,022					
0,068				0,043		0,043					
				0,024		0,024					
				0,004		0,004					
				0,016		0,016					
0,028				0,008		0,008					
0,03				0,02		0,02					
				0,037		0,037					
				0,025		0,025					
				0,004		0,004					
				0,011		0,011					

	Fig 4a				Fig 4b				Fig 4c		
H.pylori- PPI-	H.pylori- PPI+	H.pylori+ PPI	.pylori+ PPI-	PMN- PPI-	PMN- PPI+	PMN+ PPI-	PMN+ PPI+	MC low PPI-	MC low PPI+	noderate/high	oderate/high PPI+
0,027	0,01	0,181	0,022	0,181	0,01	0,055	0,022	0,018	0,022	0,181	0,047
0,054	0,022	0,018	0,047	0,027	0,022	0,015	0,008	0,054	0,008	0,027	0,01
0,082	0,005	0,055	0,008	0,018	0,005	0,017	0,078	0,082	0,022	0,055	0,078
0,005	0,013	0,015	0,078	0,054	0,019	0,111	0,014	0,018	0,005	0,015	0,014
0,018	0,057	0,066	0,014	0,082	0,013	0,015	0,008	0,05	0,008	0,066	0,14
0,028	0,009	0,024	0,008	0,005	0,057	0,097	0,009	0,025	0,009	0,024	0,014
0,01	0,014	0,017	0,009	0,018	0,014	0,073	0,14	0,016	0,057	0,017	0,019
0,004	0,008	0,133	0,14	0,01	0,008	0,038	0,009		0,008	0,133	0,013
	0,003	0,111	0,014	0,004	0,003	0,028	0,016		0,003	0,111	0,009
	0,026	0,018	0,019	0,016	0,026	0,21	0,025		0,026	0,018	0,016
	0,015	0,019	0,016		0,015	0,05	0,04		0,025	0,019	0,014
		0,015	0,007			0,024	0,02		0,014	0,015	0,007
		0,097	0,025			0,025	0,014			0,097	0,04
		0,013	0,04			0,032	0,047			0,013	0,02
		0,073	0,02			0,066	0,014			0,073	0,015
		0,12	0,014			0,024	0,007			0,12	0,025
		0,112	0,025			0,133	0,025			0,112	
		0,038				0,018				0,005	
		0,21				0,019				0,038	
		0,052				0,013				0,028	
		0,05				0,12				0,21	
		0,024				0,112				0,052	
		0,025				0,052				0,024	
		0,032				0,037				0,01	
		0,016								0,032	
		0,037								0,004	
										0,037	

CD4-	CD4+	AGS
22,22	21,36	34,07
25,54	24,02	33,04
27,62	26,78	35,05

CD4-	CD4+	AGS
0,02	0,52	0,000038
0,0026	0,013	0,000057
0,0011	0,017	0,000013
